# Crystal structure of tris­(piperidinium) hydrogen sulfate sulfate

**DOI:** 10.1107/S2056989015020538

**Published:** 2015-11-04

**Authors:** Tamara J. Lukianova, Vasyl Kinzhybalo, Adam Pietraszko

**Affiliations:** a Institute of Low Temperature and Structure Research, Polish Academy of Sciences, Okolna str. 2, PO Box 1410, 50-950 Wroclaw, Poland

**Keywords:** crystal structure, organic–inorganic hybrid, piperidinium salts, hydrogen-bonding

## Abstract

In the title molecular salt, 3C_5_H_12_N^+^·HSO_4_^−^·SO_4_^2−^, each cation adopts a chair conformation. In the crystal, the hydrogen sulfate ion is connected to the sulfate ion by a strong O—H⋯O hydrogen bond. The packing also features a number of N—H⋯O hydrogen bonds, which lead to a three-dimensional network structure. The hydrogen sulfate anion accepts four hydrogen bonds from two cations, whereas the sulfate ion, as an acceptor, binds to five separate piperidinium cations, forming seven hydrogen bonds.

## Abstract 2

A novel mixed hydrogen sulfate–sulfate piperidinium salt comprises three protonated piperidinium cations, one hydrogen sulfate anion and one sulfate anion in the asymmetric unit. Strong hydrogen bonds exist between the cations and the anions giving rise to a three-dimensional structure.

## Chemical context   

Hydrogen bonding is a powerful and versatile tool commonly used in crystal engineering to design, combine and organize individual organic mol­ecules in solids, thus creating new materials with tunable physical properties. Simple organic–inorganic salts seem to be good candidates for this purpose because of the flexibility of their special structural features such as polarity and their promising potential applications in chemistry. Not of less importance would be the use of inorganic oxyanions, which are very attractive as inorganic building blocks due to their shapes and diverse reactivity in aqueous solutions. In recent years, sulfates and hydrogen sulfates of organic bases have found applications as ionic liquids (George *et al.*, 2015 ▸). Therefore, the results of a structural study on a new molecular salt obtained from piperidine and sulfuric acid are reported here.
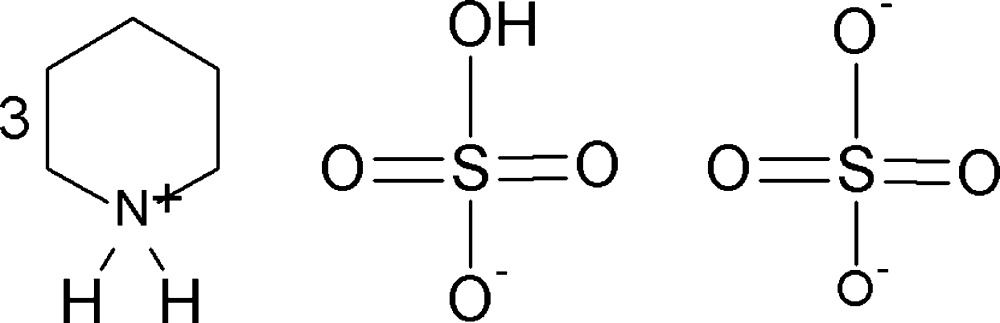


## Structural commentary   

**Figure 1:**
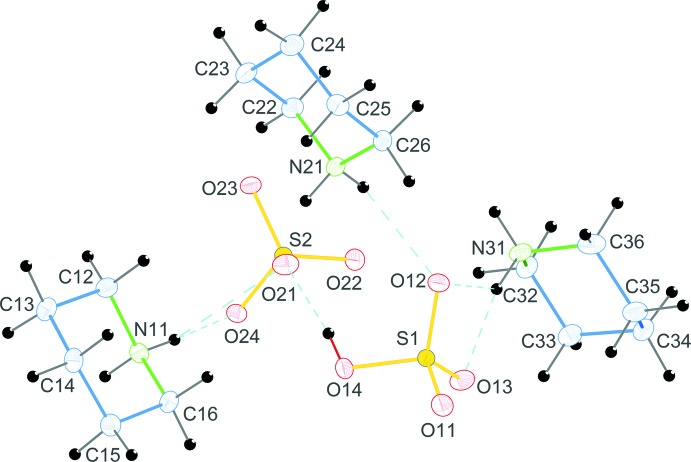
The asymmetric unit of (I), showing the atom-numbering scheme. Displacement ellipsoids are drawn at the 50% probability level. Hydrogen bonds are denoted by cyan dashed lines.

In the title compound, 3C_5_H_12_N^+^·HSO_4_^−^·SO_4_^2−^, (I), the asymmetric unit comprises three independent protonated piperidinium cations, one hydrogen sulfate anion and one sulfate anion (Fig. 1 ▸). The geometries of the three cations are similar, possessing chair conformations. The N—C and C—C bond lengths are in the ranges 1.489 (2)–1.4978 (19) Å and 1.518 (2)–1.530 (2) Å, respectively. The C—C—C, C—C—N and C—N—C angles are in the ranges 109.69 (13)–111.42 (13), 109.20 (12)–110.29 (12) and 112.01 (11)–112.30 (12)°, respectively. These values are in good agreement with those reported in the literature (Lee & Harrison, 2003 ▸). Within the cation–anion unit, the N atoms of the three piperidinium cations are connected to the O atom acceptors of the HSO_4_^−^ (O11–O14) and SO_4_^2−^ (O21–O24) anions by five N—H⋯O hydrogen bonds (Table 1 ▸). The two anions are linked *via* a short O14—H14⋯O21 hydrogen bond [2.5603 (16) Å], Figs. 1 ▸ and 2 ▸.

## Supra­molecular features   

**Figure 3:**
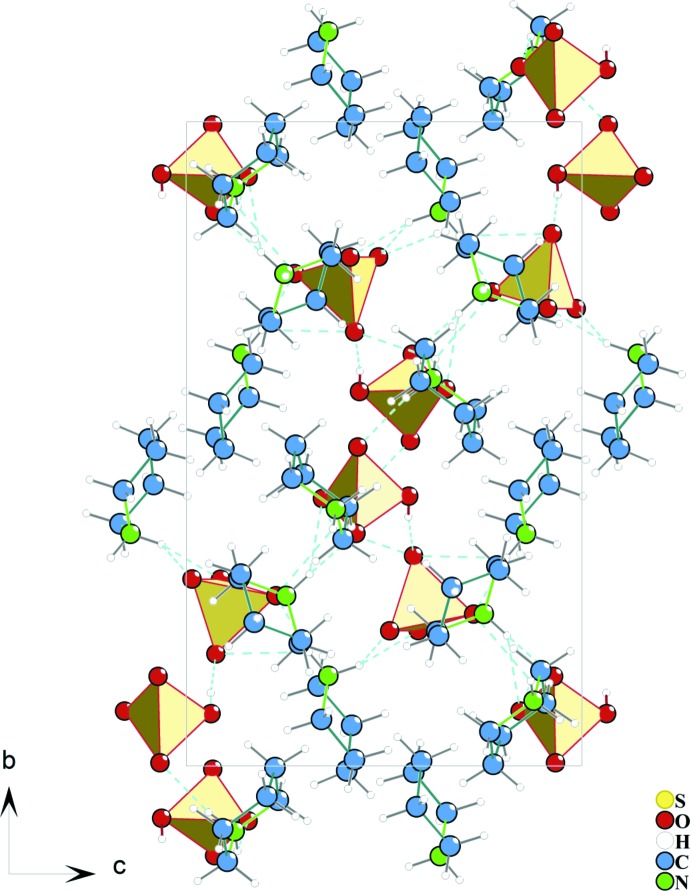
Projection of the crystal structure of (I) on the (100) plane. Hydrogen bonds are denoted by cyan dashed lines.

The crystal structure of (I) features N—H⋯O and O—H⋯O hydrogen bonds (Table 1 ▸, Fig. 1 ▸). The N atoms of the piperidinium cations are involved in hydrogen-bond formation, as donors with oxygen atoms of the sulfate and hydrogen sulfate anions. The sulfate-bound O atoms, which act as acceptors, link the organic mol­ecules through rather strong hydrogen bonds, forming a two-dimensional network of hydrogen bonds giving rise to layers parallel to (100). The hydrogen sulfate ion accepts four hydrogen bonds from three cations, whereas the sulfate ion, as an acceptor, binds to five piperidinium ions, forming seven hydrogen bonds in the overall three-dimensional structure (Fig. 3 ▸).

## Database survey   

Crystal structures of piperidinium cations with counter-anions such as hydrogen sulfide, arsenate and violurate (Smail & Sheldrick, 1973 ▸; Lee & Harrison, 2003 ▸; Kolev *et al.*, 2009 ▸) and other mixed compounds (Banerjee & Murugavel, 2004 ▸; Mohammadnezhad *et al.*, 2008 ▸; Xu *et al.*, 2009 ▸; Anderson *et al.*, 2011 ▸; Hoque & Das, 2014 ▸) have been reported.

## Synthesis and crystallization   

The title compound was prepared by the reaction between 3 ml (0.03 mol) of piperidine (Aldrich, ReagentPlus, 99%) and 3.1 ml (0.012 mol) of 30% aqueous sulfuric acid solution. The reaction mixture was continuously stirred for 15 minutes at 323 K and then allowed to cool down to room temperature. The final pH value was 2. The mixture was kept at room temperature over a period of several months, after which it was cooled in a refrigerator (*T* ≃ 278 K), giving colourless crystals of the title compound after a few weeks.

## Refinement   

Crystal data, data collection and structure refinement details are summarized in Table 2 ▸. The positions of hydrogen atoms of the amines and the hydrogen sulfate anion were initially located in difference Fourier maps but were subsequently allowed to ride in the refinement with O—H = 0.84 and N—H = 0.91 Å and with *U*_iso_(H) = 1.2*U*_eq_(N) or 1.5*U*_eq_(O). The H atom of the hydrogen sulfate anion was refined with the *SHELX* AFIX 147 instruction. Piperidinium C-bound H atoms were placed in geometrically idealized positions and also allowed to ride, with C—H = 0.99 Å and *U*_iso_(H) = 1.2*U*_eq_(C).

## Supplementary Material

Crystal structure: contains datablock(s) I. DOI: 10.1107/S2056989015020538/zs2349sup1.cif

Structure factors: contains datablock(s) I. DOI: 10.1107/S2056989015020538/zs2349Isup2.hkl

Supporting information file. DOI: 10.1107/S2056989015020538/zs2349Isup3.cml

CCDC reference: 1434209

Additional supporting information:  crystallographic information; 3D view; checkCIF report

## Figures and Tables

**Figure 2:**
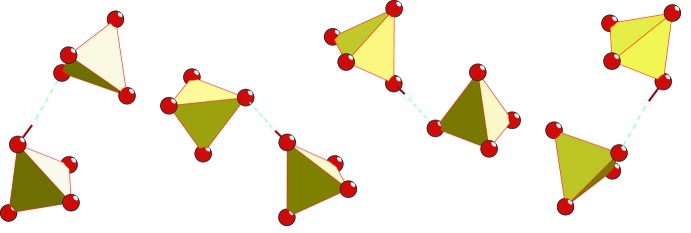
The fragments of HS_2_O_8_^3−^ anion pairs, formed from HSO_4_^−^ and SO_4_^2−^ anions *via* strong O—H⋯O hydrogen bonds (cyan dashed lines).

**Table 1:** Hydrogen-bond geometry (Å, °)

*D*—H⋯*A*	*D*—H	H⋯*A*	*D*⋯*A*	*D*—H⋯*A*
O14—H14⋯O21	0.84	1.72	2.5603 (16)	173
N21—H21*A*⋯O11^i^	0.91	1.93	2.8226 (18)	166
N21—H21*B*⋯O12	0.91	2.32	2.9096 (19)	122
N21—H21*B*⋯O24^ii^	0.91	2.47	3.0964 (18)	127
N11—H11*A*⋯O21	0.91	2.59	3.201 (2)	126
N11—H11*A*⋯O24	0.91	1.89	2.7904 (17)	171
N11—H11*B*⋯O22^iii^	0.91	2.47	3.0474 (18)	122
N11—H11*B*⋯O23^iii^	0.91	1.92	2.8039 (18)	164
N31—H31*A*⋯O12	0.91	2.41	3.0245 (18)	125
N31—H31*A*⋯O13	0.91	1.93	2.8240 (18)	167
N31—H31*B*⋯O24^ii^	0.91	1.89	2.7978 (19)	172

Symmetry codes: (i) 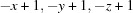; (ii) 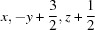; (iii) 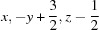.

**Table 2:** Experimental details

Crystal data	
Chemical formula	3C_5_H_12_N^+^·HSO_4_^−^·SO_4_^2−^
*M*_r_	451.60
Crystal system, space group	Monoclinic, *P*2_1_/*c*
Temperature (K)	100
*a*, *b*, *c* (Å)	10.592 (4), 17.922 (5), 11.161 (4)
β (°)	99.25 (2)
*V* (Å^3^)	2091.1 (12)
*Z*	4
Radiation type	Mo *K*α
μ (mm^−1^)	0.30
Crystal size (mm)	0.20 × 0.18 × 0.16

Data collection	
Diffractometer	Rigaku Oxford Xcalibur Atlas
Absorption correction	Analytical [*CrysAlis PRO* (Rigaku Oxford, 2015 ▸), based on expressions of Clark & Reid (1995 ▸)]
*T*_min_, *T*_max_	0.994, 0.996
No. of measured, independent and observed [*I* > 2σ(*I*)] reflections	35669, 5411, 4291
*R*_int_	0.039
(sin θ/λ)_max_ (Å^−1^)	0.691

Refinement	
*R*[*F* ^2^ > 2σ(*F* ^2^)], *wR*(*F* ^2^), *S*	0.035, 0.083, 1.03
No. of reflections	5411
No. of parameters	254
H-atom treatment	H-atom parameters constrained
Δρ_max_, Δρ_min_ (e Å^−3^)	0.33, −0.42

Computer programs: *CrysAlis PRO* (Rigaku Oxford, 2015 ▸), *SHELXS97* (Sheldrick, 2008 ▸), *SHELXL2014* (Sheldrick, 2015 ▸), *DIAMOND* (Brandenburg, 1997 ▸) and *OLEX2* (Dolomanov *et al.*, 2009 ▸).

## supplementary crystallographic information

### Crystal data

**Table:**

3C_5_H_12_N^+^·HSO_4_^−^·SO_4_^2^^−^	*F*(000) = 976
*M**_r_* = 451.60	*D*_x_ = 1.434 Mg m^−^^3^
Monoclinic, *P*2_1_/*c*	Mo *K*α radiation, λ = 0.71073 Å
*a* = 10.592 (4) Å	Cell parameters from 12552 reflections
*b* = 17.922 (5) Å	θ = 2.2–29.4°
*c* = 11.161 (4) Å	µ = 0.30 mm^−^^1^
β = 99.25 (2)°	*T* = 100 K
*V* = 2091.1 (12) Å^3^	Block, colourless
*Z* = 4	0.20 × 0.18 × 0.16 mm

### Data collection

**Table:**

Rigaku Oxford Xcalibur Atlas diffractometer	5411 independent reflections
Radiation source: fine-focus sealed X-ray tube, Enhance (Mo) X-ray source	4291 reflections with *I* > 2σ(*I*)
Graphite monochromator	*R*_int_ = 0.039
Detector resolution: 10.6249 pixels mm^-1^	θ_max_ = 29.4°, θ_min_ = 2.7°
ω scans	*h* = −14→14
Absorption correction: analytical [*CrysAlis PRO* (Rigaku Oxford, 2015), based on expressions of Clark & Reid (1995)]	*k* = −23→24
*T*_min_ = 0.994, *T*_max_ = 0.996	*l* = −15→15
35669 measured reflections	

### Refinement

**Table:**

Refinement on *F*^2^	0 restraints
Least-squares matrix: full	Hydrogen site location: inferred from neighbouring sites
*R*[*F*^2^ > 2σ(*F*^2^)] = 0.035	H-atom parameters constrained
*wR*(*F*^2^) = 0.083	*w* = 1/[σ^2^(*F*_o_^2^) + (0.0343*P*)^2^ + 1.1863*P*] where *P* = (*F*_o_^2^ + 2*F*_c_^2^)/3
*S* = 1.03	(Δ/σ)_max_ = 0.001
5411 reflections	Δρ_max_ = 0.33 e Å^−^^3^
254 parameters	Δρ_min_ = −0.42 e Å^−^^3^

### Special details

**Table:**

Geometry. All e.s.d.'s (except the e.s.d. in the dihedral angle between two l.s. planes) are estimated using the full covariance matrix. The cell e.s.d.'s are taken into account individually in the estimation of e.s.d.'s in distances, angles and torsion angles; correlations between e.s.d.'s in cell parameters are only used when they are defined by crystal symmetry. An approximate (isotropic) treatment of cell e.s.d.'s is used for estimating e.s.d.'s involving l.s. planes.

### Fractional atomic coordinates and isotropic or equivalent isotropic displacement parameters (Å^2^)

**Table:**

	*x*	*y*	*z*	*U*_iso_*/*U*_eq_	
S1	0.66217 (3)	0.57717 (2)	0.56541 (3)	0.01118 (9)	
S2	0.39851 (3)	0.75550 (2)	0.40181 (3)	0.01134 (9)	
O12	0.57890 (10)	0.58513 (6)	0.65614 (9)	0.0155 (2)	
O21	0.39138 (10)	0.67389 (6)	0.42549 (10)	0.0183 (2)	
O14	0.57685 (10)	0.58114 (6)	0.43754 (10)	0.0175 (2)	
H14	0.5194	0.6134	0.4385	0.026*	
O11	0.72505 (10)	0.50505 (6)	0.56684 (10)	0.0174 (2)	
O13	0.75266 (10)	0.63911 (6)	0.57133 (10)	0.0180 (2)	
O22	0.50129 (11)	0.79014 (7)	0.48587 (10)	0.0222 (3)	
C25	0.19519 (15)	0.50258 (8)	0.72404 (14)	0.0154 (3)	
H25A	0.2000	0.4735	0.8001	0.018*	
H25B	0.1955	0.4669	0.6563	0.018*	
N21	0.30210 (12)	0.60129 (7)	0.62212 (11)	0.0127 (3)	
H21A	0.3029	0.5718	0.5559	0.015*	
H21B	0.3716	0.6318	0.6293	0.015*	
O23	0.27443 (10)	0.78993 (6)	0.41375 (9)	0.0142 (2)	
O24	0.42147 (10)	0.76415 (6)	0.27366 (9)	0.0135 (2)	
N11	0.32098 (12)	0.63977 (7)	0.14102 (11)	0.0131 (3)	
H11A	0.3570	0.6768	0.1909	0.016*	
H11B	0.3156	0.6560	0.0631	0.016*	
N31	0.67984 (12)	0.73600 (7)	0.74774 (12)	0.0159 (3)	
H31A	0.6901	0.7023	0.6890	0.019*	
H31B	0.5947	0.7394	0.7510	0.019*	
C14	0.21075 (15)	0.49250 (8)	0.09492 (14)	0.0164 (3)	
H14A	0.2125	0.4710	0.1768	0.020*	
H14B	0.1725	0.4551	0.0345	0.020*	
C26	0.31146 (14)	0.55327 (8)	0.73257 (13)	0.0138 (3)	
H26A	0.3169	0.5851	0.8057	0.017*	
H26B	0.3901	0.5226	0.7403	0.017*	
C23	0.06623 (15)	0.59747 (9)	0.59190 (15)	0.0176 (3)	
H23A	0.0622	0.5662	0.5183	0.021*	
H23B	−0.0121	0.6285	0.5828	0.021*	
C16	0.40461 (14)	0.57238 (8)	0.15854 (14)	0.0152 (3)	
H16A	0.4139	0.5557	0.2441	0.018*	
H16B	0.4907	0.5850	0.1406	0.018*	
C22	0.18310 (14)	0.64782 (8)	0.60349 (14)	0.0155 (3)	
H22A	0.1799	0.6782	0.5290	0.019*	
H22B	0.1837	0.6821	0.6731	0.019*	
C24	0.07120 (15)	0.54724 (9)	0.70327 (15)	0.0189 (3)	
H24A	0.0655	0.5782	0.7757	0.023*	
H24B	−0.0027	0.5127	0.6912	0.023*	
C12	0.18991 (14)	0.62418 (9)	0.16711 (14)	0.0160 (3)	
H12A	0.1374	0.6701	0.1552	0.019*	
H12B	0.1946	0.6080	0.2525	0.019*	
C33	0.86970 (15)	0.80450 (9)	0.70869 (15)	0.0180 (3)	
H33A	0.8828	0.7698	0.6428	0.022*	
H33B	0.9023	0.8541	0.6895	0.022*	
C35	0.89016 (16)	0.70228 (10)	0.86207 (16)	0.0217 (4)	
H35A	0.9040	0.6641	0.8014	0.026*	
H35B	0.9357	0.6860	0.9422	0.026*	
C15	0.34720 (15)	0.50991 (8)	0.07536 (14)	0.0161 (3)	
H15A	0.4007	0.4646	0.0915	0.019*	
H15B	0.3466	0.5247	−0.0102	0.019*	
C13	0.12870 (15)	0.56320 (9)	0.08232 (15)	0.0169 (3)	
H13A	0.1182	0.5812	−0.0026	0.020*	
H13B	0.0428	0.5515	0.1015	0.020*	
C32	0.72742 (14)	0.81012 (8)	0.71454 (15)	0.0167 (3)	
H32A	0.7129	0.8477	0.7758	0.020*	
H32B	0.6804	0.8260	0.6348	0.020*	
C36	0.74807 (16)	0.70877 (9)	0.86681 (16)	0.0205 (3)	
H36A	0.7138	0.6595	0.8854	0.025*	
H36B	0.7343	0.7440	0.9318	0.025*	
C34	0.94462 (15)	0.77687 (10)	0.82831 (15)	0.0207 (3)	
H34A	0.9390	0.8139	0.8930	0.025*	
H34B	1.0358	0.7708	0.8205	0.025*	

### Atomic displacement parameters (Å^2^)

**Table:**

	*U*^11^	*U*^22^	*U*^33^	*U*^12^	*U*^13^	*U*^23^
S1	0.01134 (17)	0.01084 (17)	0.01124 (17)	0.00075 (13)	0.00142 (13)	−0.00154 (13)
S2	0.01054 (17)	0.01264 (17)	0.01101 (17)	0.00085 (13)	0.00228 (13)	−0.00016 (13)
O12	0.0153 (5)	0.0173 (5)	0.0147 (5)	0.0002 (4)	0.0049 (4)	−0.0014 (4)
O21	0.0207 (6)	0.0144 (5)	0.0214 (6)	0.0051 (4)	0.0077 (5)	0.0061 (4)
O14	0.0175 (6)	0.0209 (6)	0.0127 (5)	0.0071 (4)	−0.0014 (4)	−0.0035 (4)
O11	0.0208 (6)	0.0132 (5)	0.0174 (6)	0.0054 (4)	0.0013 (5)	−0.0018 (4)
O13	0.0147 (5)	0.0157 (5)	0.0246 (6)	−0.0029 (4)	0.0063 (5)	−0.0038 (4)
O22	0.0144 (5)	0.0339 (7)	0.0180 (6)	−0.0035 (5)	0.0013 (5)	−0.0088 (5)
C25	0.0189 (8)	0.0125 (7)	0.0154 (7)	−0.0006 (6)	0.0046 (6)	0.0019 (6)
N21	0.0109 (6)	0.0133 (6)	0.0140 (6)	−0.0009 (5)	0.0023 (5)	0.0015 (5)
O23	0.0130 (5)	0.0152 (5)	0.0152 (5)	0.0029 (4)	0.0043 (4)	0.0004 (4)
O24	0.0147 (5)	0.0141 (5)	0.0124 (5)	0.0001 (4)	0.0041 (4)	0.0008 (4)
N11	0.0151 (6)	0.0129 (6)	0.0114 (6)	−0.0017 (5)	0.0023 (5)	0.0004 (5)
N31	0.0110 (6)	0.0143 (6)	0.0231 (7)	−0.0014 (5)	0.0046 (5)	−0.0073 (5)
C14	0.0169 (7)	0.0141 (7)	0.0168 (8)	−0.0024 (6)	−0.0017 (6)	−0.0003 (6)
C26	0.0139 (7)	0.0143 (7)	0.0127 (7)	0.0006 (6)	0.0008 (6)	0.0028 (6)
C23	0.0122 (7)	0.0232 (8)	0.0177 (8)	0.0023 (6)	0.0027 (6)	0.0040 (6)
C16	0.0128 (7)	0.0157 (7)	0.0163 (7)	0.0006 (6)	0.0000 (6)	0.0016 (6)
C22	0.0159 (7)	0.0140 (7)	0.0168 (7)	0.0040 (6)	0.0034 (6)	0.0034 (6)
C24	0.0151 (7)	0.0226 (8)	0.0200 (8)	−0.0014 (6)	0.0058 (6)	0.0039 (6)
C12	0.0138 (7)	0.0170 (7)	0.0177 (8)	−0.0006 (6)	0.0042 (6)	−0.0001 (6)
C33	0.0148 (7)	0.0159 (7)	0.0236 (8)	−0.0010 (6)	0.0044 (7)	0.0003 (6)
C35	0.0195 (8)	0.0271 (9)	0.0196 (8)	0.0088 (7)	0.0061 (7)	0.0044 (7)
C15	0.0168 (8)	0.0148 (7)	0.0162 (7)	0.0008 (6)	0.0010 (6)	−0.0002 (6)
C13	0.0132 (7)	0.0173 (7)	0.0194 (8)	−0.0015 (6)	0.0001 (6)	0.0006 (6)
C32	0.0140 (7)	0.0147 (7)	0.0208 (8)	0.0008 (6)	0.0012 (6)	−0.0004 (6)
C36	0.0210 (8)	0.0185 (8)	0.0242 (9)	0.0035 (6)	0.0106 (7)	0.0043 (6)
C34	0.0124 (7)	0.0273 (9)	0.0217 (8)	−0.0013 (6)	0.0009 (6)	−0.0050 (7)

### Geometric parameters (Å, º)

**Table:**

S1—O12	1.4529 (12)	C23—H23B	0.9900
S1—O14	1.5633 (12)	C23—C22	1.520 (2)
S1—O11	1.4530 (11)	C23—C24	1.529 (2)
S1—O13	1.4612 (11)	C16—H16A	0.9900
S2—O21	1.4904 (12)	C16—H16B	0.9900
S2—O22	1.4569 (12)	C16—C15	1.518 (2)
S2—O23	1.4773 (11)	C22—H22A	0.9900
S2—O24	1.4969 (12)	C22—H22B	0.9900
O14—H14	0.8400	C24—H24A	0.9900
C25—H25A	0.9900	C24—H24B	0.9900
C25—H25B	0.9900	C12—H12A	0.9900
C25—C26	1.521 (2)	C12—H12B	0.9900
C25—C24	1.523 (2)	C12—C13	1.521 (2)
N21—H21A	0.9100	C33—H33A	0.9900
N21—H21B	0.9100	C33—H33B	0.9900
N21—C26	1.4936 (19)	C33—C32	1.522 (2)
N21—C22	1.4978 (19)	C33—C34	1.522 (2)
N11—H11A	0.9100	C35—H35A	0.9900
N11—H11B	0.9100	C35—H35B	0.9900
N11—C16	1.4920 (19)	C35—C36	1.519 (2)
N11—C12	1.4900 (19)	C35—C34	1.527 (2)
N31—H31A	0.9100	C15—H15A	0.9900
N31—H31B	0.9100	C15—H15B	0.9900
N31—C32	1.489 (2)	C13—H13A	0.9900
N31—C36	1.489 (2)	C13—H13B	0.9900
C14—H14A	0.9900	C32—H32A	0.9900
C14—H14B	0.9900	C32—H32B	0.9900
C14—C15	1.528 (2)	C36—H36A	0.9900
C14—C13	1.530 (2)	C36—H36B	0.9900
C26—H26A	0.9900	C34—H34A	0.9900
C26—H26B	0.9900	C34—H34B	0.9900
C23—H23A	0.9900		
			
O12—S1—O14	107.77 (7)	N21—C22—C23	109.68 (12)
O12—S1—O11	114.10 (7)	N21—C22—H22A	109.7
O12—S1—O13	111.17 (7)	N21—C22—H22B	109.7
O11—S1—O14	104.31 (6)	C23—C22—H22A	109.7
O11—S1—O13	112.28 (7)	C23—C22—H22B	109.7
O13—S1—O14	106.58 (7)	H22A—C22—H22B	108.2
O21—S2—O24	106.96 (6)	C25—C24—C23	110.44 (13)
O22—S2—O21	110.98 (7)	C25—C24—H24A	109.6
O22—S2—O23	110.32 (7)	C25—C24—H24B	109.6
O22—S2—O24	110.60 (7)	C23—C24—H24A	109.6
O23—S2—O21	108.83 (6)	C23—C24—H24B	109.6
O23—S2—O24	109.05 (6)	H24A—C24—H24B	108.1
S1—O14—H14	109.5	N11—C12—H12A	109.8
H25A—C25—H25B	108.0	N11—C12—H12B	109.8
C26—C25—H25A	109.3	N11—C12—C13	109.30 (12)
C26—C25—H25B	109.3	H12A—C12—H12B	108.3
C26—C25—C24	111.42 (13)	C13—C12—H12A	109.8
C24—C25—H25A	109.3	C13—C12—H12B	109.8
C24—C25—H25B	109.3	H33A—C33—H33B	108.0
H21A—N21—H21B	107.9	C32—C33—H33A	109.4
C26—N21—H21A	109.2	C32—C33—H33B	109.4
C26—N21—H21B	109.2	C34—C33—H33A	109.4
C26—N21—C22	112.22 (11)	C34—C33—H33B	109.4
C22—N21—H21A	109.2	C34—C33—C32	111.35 (14)
C22—N21—H21B	109.2	H35A—C35—H35B	108.0
H11A—N11—H11B	107.9	C36—C35—H35A	109.5
C16—N11—H11A	109.2	C36—C35—H35B	109.5
C16—N11—H11B	109.2	C36—C35—C34	110.94 (13)
C12—N11—H11A	109.2	C34—C35—H35A	109.5
C12—N11—H11B	109.2	C34—C35—H35B	109.5
C12—N11—C16	112.01 (11)	C14—C15—H15A	109.4
H31A—N31—H31B	107.9	C14—C15—H15B	109.4
C32—N31—H31A	109.1	C16—C15—C14	111.00 (13)
C32—N31—H31B	109.1	C16—C15—H15A	109.4
C32—N31—C36	112.30 (12)	C16—C15—H15B	109.4
C36—N31—H31A	109.1	H15A—C15—H15B	108.0
C36—N31—H31B	109.1	C14—C13—H13A	109.4
H14A—C14—H14B	108.1	C14—C13—H13B	109.4
C15—C14—H14A	109.5	C12—C13—C14	111.03 (13)
C15—C14—H14B	109.5	C12—C13—H13A	109.4
C15—C14—C13	110.74 (12)	C12—C13—H13B	109.4
C13—C14—H14A	109.5	H13A—C13—H13B	108.0
C13—C14—H14B	109.5	N31—C32—C33	109.20 (12)
C25—C26—H26A	109.6	N31—C32—H32A	109.8
C25—C26—H26B	109.6	N31—C32—H32B	109.8
N21—C26—C25	110.29 (12)	C33—C32—H32A	109.8
N21—C26—H26A	109.6	C33—C32—H32B	109.8
N21—C26—H26B	109.6	H32A—C32—H32B	108.3
H26A—C26—H26B	108.1	N31—C36—C35	109.60 (13)
H23A—C23—H23B	108.0	N31—C36—H36A	109.8
C22—C23—H23A	109.4	N31—C36—H36B	109.8
C22—C23—H23B	109.4	C35—C36—H36A	109.8
C22—C23—C24	111.05 (13)	C35—C36—H36B	109.8
C24—C23—H23A	109.4	H36A—C36—H36B	108.2
C24—C23—H23B	109.4	C33—C34—C35	109.69 (13)
N11—C16—H16A	109.6	C33—C34—H34A	109.7
N11—C16—H16B	109.6	C33—C34—H34B	109.7
N11—C16—C15	110.24 (12)	C35—C34—H34A	109.7
H16A—C16—H16B	108.1	C35—C34—H34B	109.7
C15—C16—H16A	109.6	H34A—C34—H34B	108.2
C15—C16—H16B	109.6		
			
N11—C16—C15—C14	−55.57 (16)	C12—N11—C16—C15	59.41 (16)
N11—C12—C13—C14	57.09 (17)	C15—C14—C13—C12	−54.79 (17)
C26—C25—C24—C23	54.49 (17)	C13—C14—C15—C16	53.79 (17)
C26—N21—C22—C23	−58.61 (16)	C32—N31—C36—C35	−59.33 (17)
C16—N11—C12—C13	−59.86 (16)	C32—C33—C34—C35	55.93 (17)
C22—N21—C26—C25	57.84 (16)	C36—N31—C32—C33	59.01 (17)
C22—C23—C24—C25	−55.39 (18)	C36—C35—C34—C33	−55.78 (18)
C24—C25—C26—N21	−55.37 (16)	C34—C33—C32—N31	−57.02 (17)
C24—C23—C22—N21	56.89 (17)	C34—C35—C36—N31	57.05 (18)

### Hydrogen-bond geometry (Å, º)

**Table:**

*D*—H···*A*	*D*—H	H···*A*	*D*···*A*	*D*—H···*A*
O14—H14···O21	0.84	1.72	2.5603 (16)	173
N21—H21*A*···O11^i^	0.91	1.93	2.8226 (18)	166
N21—H21*B*···O12	0.91	2.32	2.9096 (19)	122
N21—H21*B*···O24^ii^	0.91	2.47	3.0964 (18)	127
N11—H11*A*···O21	0.91	2.59	3.201 (2)	126
N11—H11*A*···O24	0.91	1.89	2.7904 (17)	171
N11—H11*B*···O22^iii^	0.91	2.47	3.0474 (18)	122
N11—H11*B*···O23^iii^	0.91	1.92	2.8039 (18)	164
N31—H31*A*···O12	0.91	2.41	3.0245 (18)	125
N31—H31*A*···O13	0.91	1.93	2.8240 (18)	167
N31—H31*B*···O24^ii^	0.91	1.89	2.7978 (19)	172

Symmetry codes: (i) −*x*+1, −*y*+1, −*z*+1; (ii) *x*, −*y*+3/2, *z*+1/2; (iii) *x*, −*y*+3/2, *z*−1/2.
